# Autonomous exoskeleton reduces metabolic cost of human walking

**DOI:** 10.1186/1743-0003-11-151

**Published:** 2014-11-03

**Authors:** Luke M Mooney, Elliott J Rouse, Hugh M Herr

**Affiliations:** Center for Extreme Bionics, MIT Media Lab, Massachusetts Institute of Technology, Cambridge, USA

**Keywords:** Exoskeleton, Autonomous, Metabolic, Power, Walking

## Abstract

**Background:**

Passive exoskeletons that assist with human locomotion are often lightweight and compact, but are unable to provide net mechanical power to the exoskeletal wearer. In contrast, powered exoskeletons often provide biologically appropriate levels of mechanical power, but the size and mass of their actuator/power source designs often lead to heavy and unwieldy devices. In this study, we extend the design and evaluation of a lightweight and powerful autonomous exoskeleton evaluated for loaded walking in (J Neuroeng Rehab 11:80, 2014) to the case of unloaded walking conditions.

**Findings:**

The metabolic energy consumption of seven study participants (85 ± 12 kg body mass) was measured while walking on a level treadmill at 1.4 m/s. Testing conditions included not wearing the exoskeleton and wearing the exoskeleton, in both powered and unpowered modes. When averaged across the gait cycle, the autonomous exoskeleton applied a mean positive mechanical power of 26 ± 1 W (13 W per ankle) with 2.12 kg of added exoskeletal foot-shank mass (1.06 kg per leg). Use of the leg exoskeleton significantly reduced the metabolic cost of walking by 35 ± 13 W, which was an improvement of 10 ± 3% (*p* = 0.023) relative to the control condition of not wearing the exoskeleton.

**Conclusions:**

The results of this study highlight the advantages of developing lightweight and powerful exoskeletons that can comfortably assist the body during walking.

**Electronic supplementary material:**

The online version of this article (doi:10.1186/1743-0003-11-151) contains supplementary material, which is available to authorized users.

## Findings

### Introduction

For over a century, researchers have designed stand-alone, or autonomous, exoskeletal mechanisms with the hope of amplifying human ambulatory performance. Previous exoskeletal research has resulted in many interesting designs and much has been learned regarding human-machine interaction [1–5]. However, the development of an effective autonomous exoskeleton remained a challenge until very recently. Our recent publication described an autonomous ankle exoskeleton that reduced the metabolic burden to walk by 8% while carrying load, when compared to not wearing the device [6]. This previous investigation provided important insight into the feasibility of autonomous exoskeletons for loaded walking, but not the more common case of unloaded walking.

In the present study, we refine the previously published exoskeletal design described in [6] and further evaluate its impact on the metabolic consumption for unloaded, level ground walking. In our previous study [6] we presented the Augmentation Factor (AF), a metric that predicts the metabolic impact of a device based on exoskeletal mass and power characteristics. Based on the AF, we hypothesized that the revised exoskeletal design would significantly reduce the metabolic energy consumed by a human wearer for normal, unloaded walking. We evaluated this hypothesis by measuring the metabolic energy consumption of seven study participants walking on a level treadmill at 1.4 m/s. Testing conditions included participants not wearing the exoskeleton and wearing the exoskeleton, both for powered on and powered off exoskeletal modes.

## Methods

**Figure 1:**
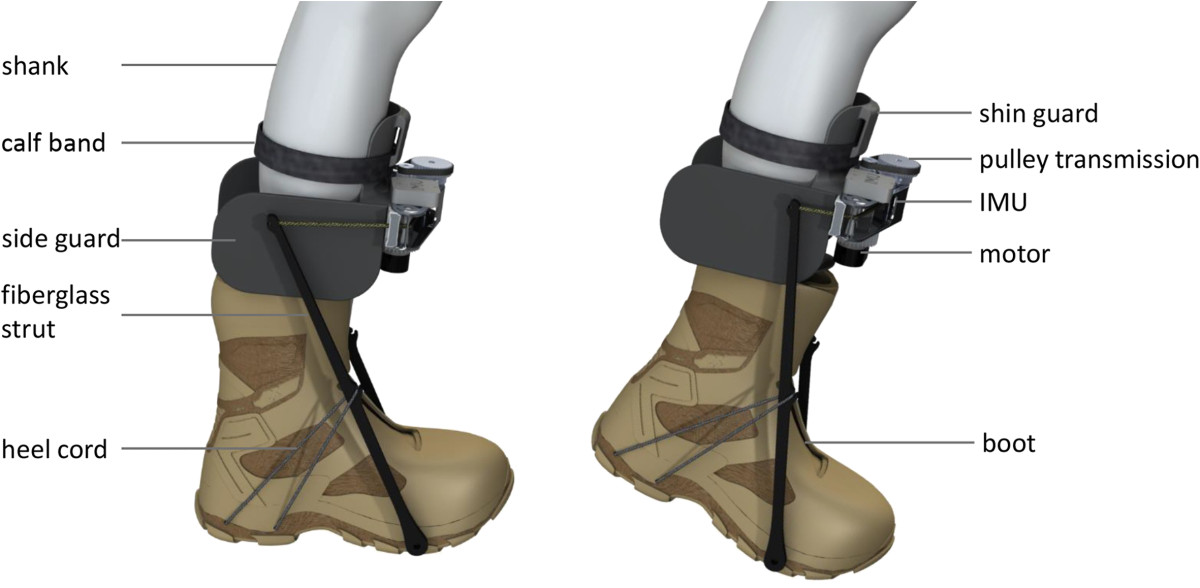
**Autonomous leg exoskeleton.** The posterior protrusion of the device was reduced compared to the former exoskeleton [6] by using shorter struts and increasing the length of the heel cord. Further, side guards were added to eliminate strut rubbing against the calves during walking.

The autonomous exoskeletal design of this investigation is similar to the previously published device in [6], but with minor modifications implemented to improve overall design performance. To reduce mass, the foot switches (103 g each) were removed from each boot and replaced with a gyroscope on each actuator (model: LPY550ALTR, STMicroelectronics, Geneva, CH). The struts were also shortened in order to both reduce mass and their posterior protrusion. The effective strut moment arm from the ankle joint was reduced from 300 mm to 230 mm. Posterior protrusion of the struts were further reduced by increasing the acute angle formed by the struts and the bottom of the boots (Figure 1). Shortening and realignment of the struts resulted in their proximal ends being directly against the medial and lateral sides of the wearer’s calves. To eliminate direct interference with the biological leg, side guards were added to the actuator structure to effectively separate the struts from the leg. Without further modification, the shorter moment arm would have resulted in a reduced transmission ratio and electrical efficiency. To mitigate this effect, the pulley transmission ratio was increased from 13:8 to 44:14. These changes resulted in an overall transmission ratio of approximately 160, which is a 28% increase from the previously published device [6]. Overall, the aforementioned modifications resulted in a lighter device, a more compact form factor and a higher overall transmission ratio. The exoskeleton mass distribution is shown in Table 1.

**Table 1:** **Exoskeleton component mass distribution**

Part	Mass (g)	Location
Strut assembly	190 (×2)	Foot & Shank
Winch actuator	870 (×2)	Shank
Motor Controllers	660	Waist
Batteries	820	Back
**Total**	**3600**	

Components that have both a right and left leg version are denoted with (×2), and the shown component mass is only for one exoskeletal leg.

The onboard controllers and gyroscopes implemented an adaptive timing strategy to consistently generate positive power during late stance powered plantar flexion and zero torque during swing. The integrated gyroscope was sampled at 250 Hz and filtered with a 2^nd^ order, 6 Hz low pass Butterworth filter. Subsequently, the signal was used to estimate the shank’s angular velocity in the sagittal plane for the detection of walking gait phases. Specifically, heel contact after a swing phase was estimated to occur when a positive shank velocity (leg protraction) was continuously detected for 220 ms or longer, followed by an angular velocity zero-crossing resulting in the velocity first becoming negative (the initiation of leg retraction). At heel strike, the adaptive timer started (400–500 ms) and the exoskeleton applied a slight plantar flexion torque to maintain tension in the cord. The end of the timer signaled the beginning of stance phase power assistance. Power assistance was achieved by applying a parabolic voltage profile to the motor over 150 ms. Subsequent to power assistance, the controller automatically entered swing phase. At this time, the controller quickly released the cord over 125 ms to provide slack so as not to impede the user. Finally, the controller entered an idle state after providing slack until the next ipsilateral heel strike was detected.

Timing and power magnitude were adjusted to align with reference biological power profiles [7]. The mechanical power applied by the exoskeleton was estimated with a linear motor model and an actuator efficiency characterization [6]. Using the period of the previous step, the location and magnitude of the peak power were estimated. The adaptive timer duration was incrementally adjusted such that the peak power aligned with 53% gait cycle [7]. Similarly, the amplitude of the mechanical power profile was continuously adjusted to maintain a normalized peak power of 2.3 W/kg, 70% of the peak ankle power typically reported for normal walking [7]. This mechanical power level was chosen to prevent the thermal overload of the motors while testing with participants that had greater mass.

The metabolic effect of the autonomous powered exoskeleton on level ground walking was experimentally determined using seven study participants (6 male; 1 female; 85 ± 12 kg body mass; 180 ± 9 cm stature; 26 ± 5 years old; mean ± standard deviation). Participants walked on a treadmill at 1.4 m/s, approximately equal to the average adult walking speed [8]. All participants were healthy and exhibited no gait abnormalities. This study was approved by the MIT Committee on the Use of Humans as Experimental Subjects, and informed consent was obtained from experimental participants. A portable pulmonary gas exchange measurement instrument (model: K4b2, COSMED, Rome, IT) was worn by the participants during four walking trials and two standing trials. The tests began with the participants standing for 6 minutes, in order to obtain a resting metabolic rate. Then, each participant walked for 10 minutes without the exoskeleton, 20 minutes while wearing the exoskeleton in a powered on state^a^, 20 minutes with the exoskeleton in a powered off state, and once again 10 minutes without the exoskeleton.

Metabolic rate was calculated from oxygen consumption and carbon dioxide production rates measured by the portable pulmonary gas exchange measurement unit. The average rates of the last five minutes of each trial were converted into metabolic power using the equation developed by Brockway et al. [9]. The metabolic rate of standing was subtracted from the gross metabolic rates of walking in order to obtain the net metabolic costs of walking. The net metabolic rates measured from the two control trials were averaged and compared to the net metabolic rates of the exoskeleton trials.

## Results

**Figure 2:**
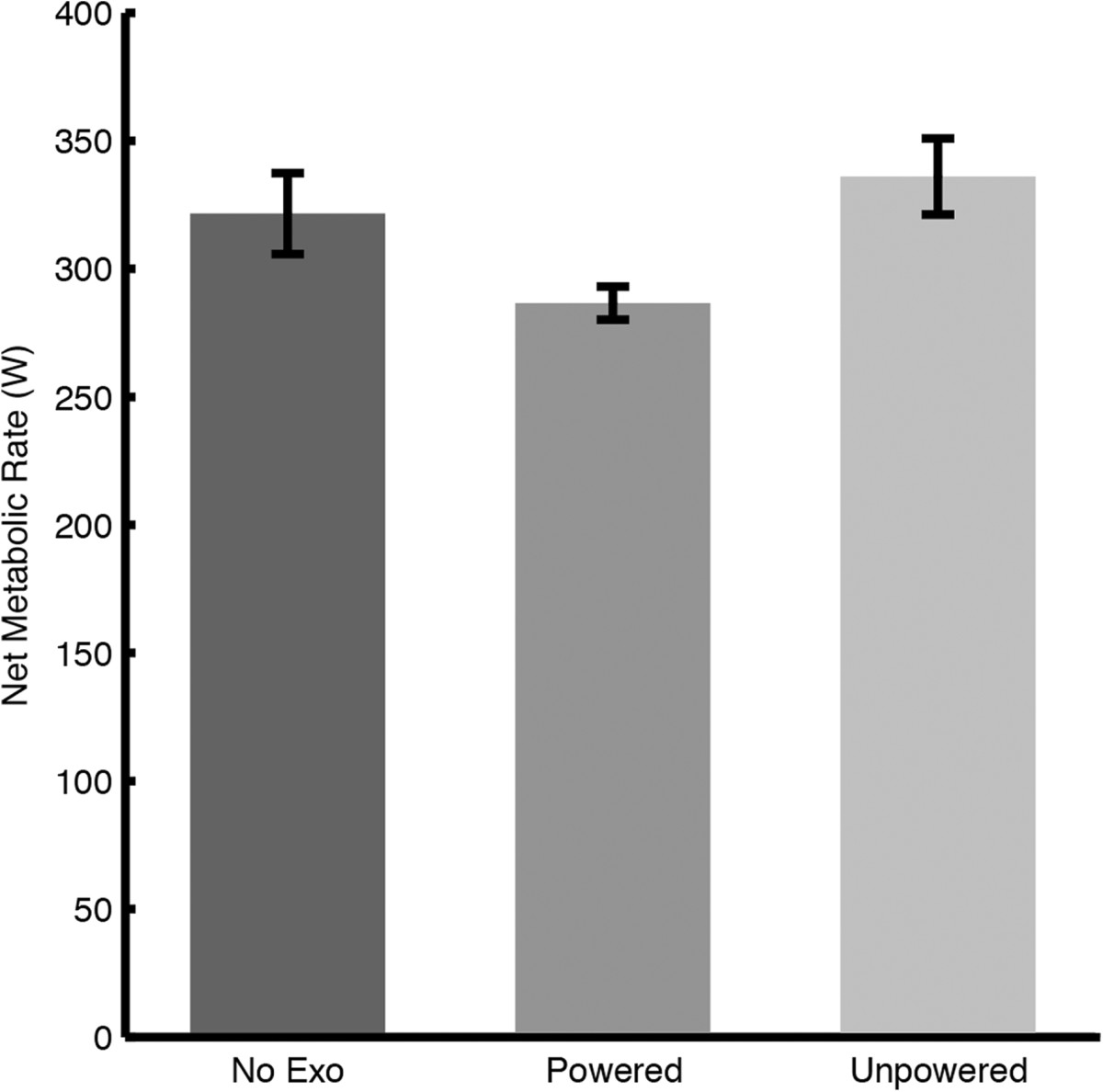
**Metabolic comparison of walking trials.** The net metabolic cost of walking is shown without the exoskeleton, with the powered exoskeleton, and with the unpowered exoskeleton. Standard error bars are shown for each experimental condition.

**Figure 3:**
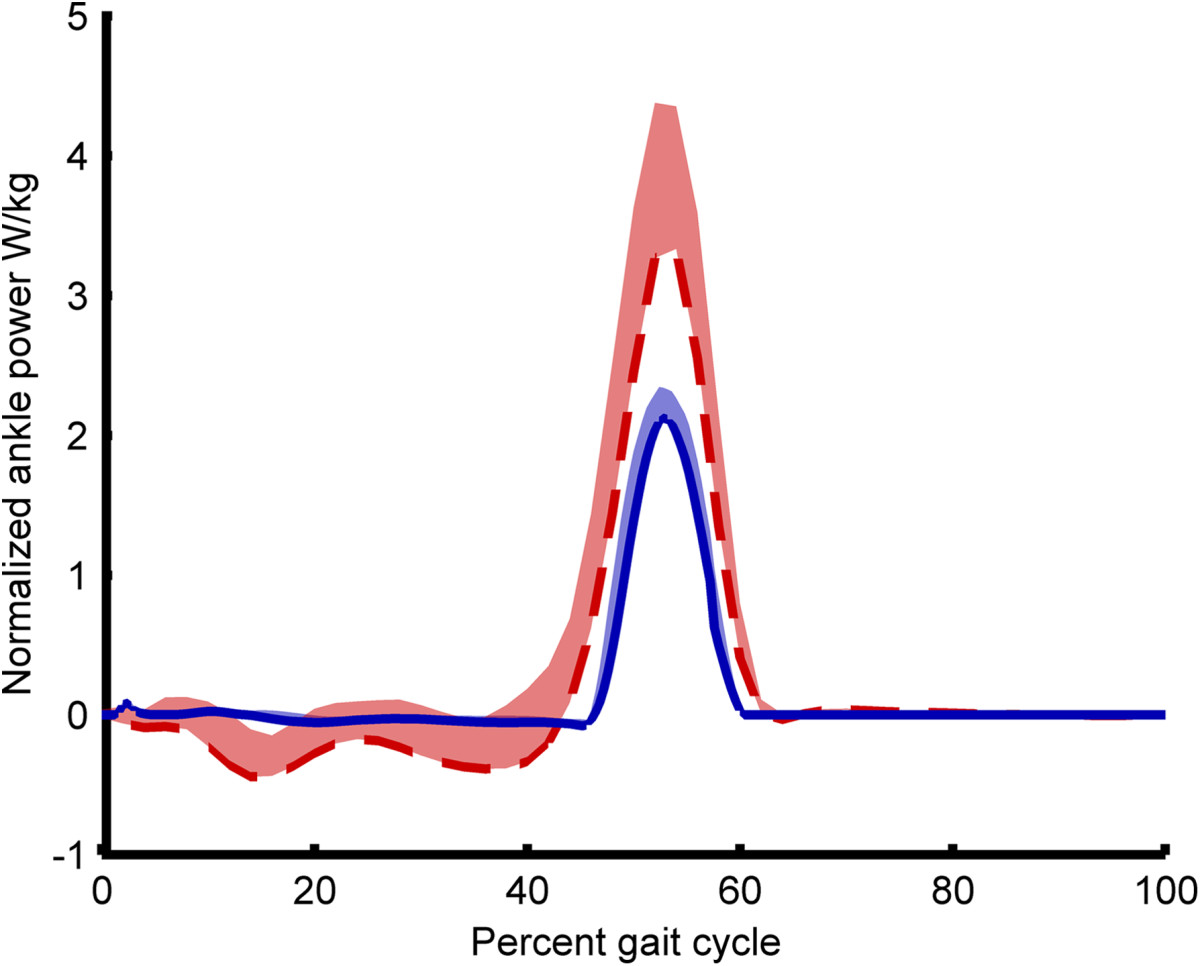
**Exoskeletal mechanical power.** Inter-subject mean exoskeletal ankle power provided by only the exoskeleton is shown (solid blue) throughout a single gait cycle. Power is normalized by body mass with standard deviation shown in translucent. For comparison, the mechanical power provided by only the biological ankle joint is shown (dashed red) for normal walking, acquired from a reference dataset [7].

The autonomous exoskeleton significantly reduced the metabolic power required to walk at 1.4 m/s. The net metabolic cost of walking without the exoskeleton was 3.82 ± 0.23 W/kg (mean ± standard error). The mean metabolic cost of walking with the powered and unpowered exoskeleton was 3.43 ± 0.23 W/kg and 4.01 ± 0.30 W/kg, respectively. Use of the autonomous leg exoskeleton significantly reduced the metabolic cost of walking by 35 ± 13 W, an improvement of 10 ± 3% (p = 0.023) relative to the control condition (Figure 2). All seven subjects showed improvements ranging from 1% to 22%. Compared to the unpowered condition, the powered exoskeleton reduced the metabolic cost of walking by 49 ± 10 W, an improvement of 14 ± 2% (p <0.001). The powered exoskeleton generated a mean positive mechanical power of 26 ± 1 W (13 ± 0.7 W per ankle) during powered plantar flexion (Figure 3). The mean electrical power was measured to be 45 ± 1 W. If one hundred percent of the battery’s energy was used, or 432 kJ, then the exoskeleton would have a battery life of 2.7 hours, or 13 km at 1.4 m/s.

**Figure 4:**
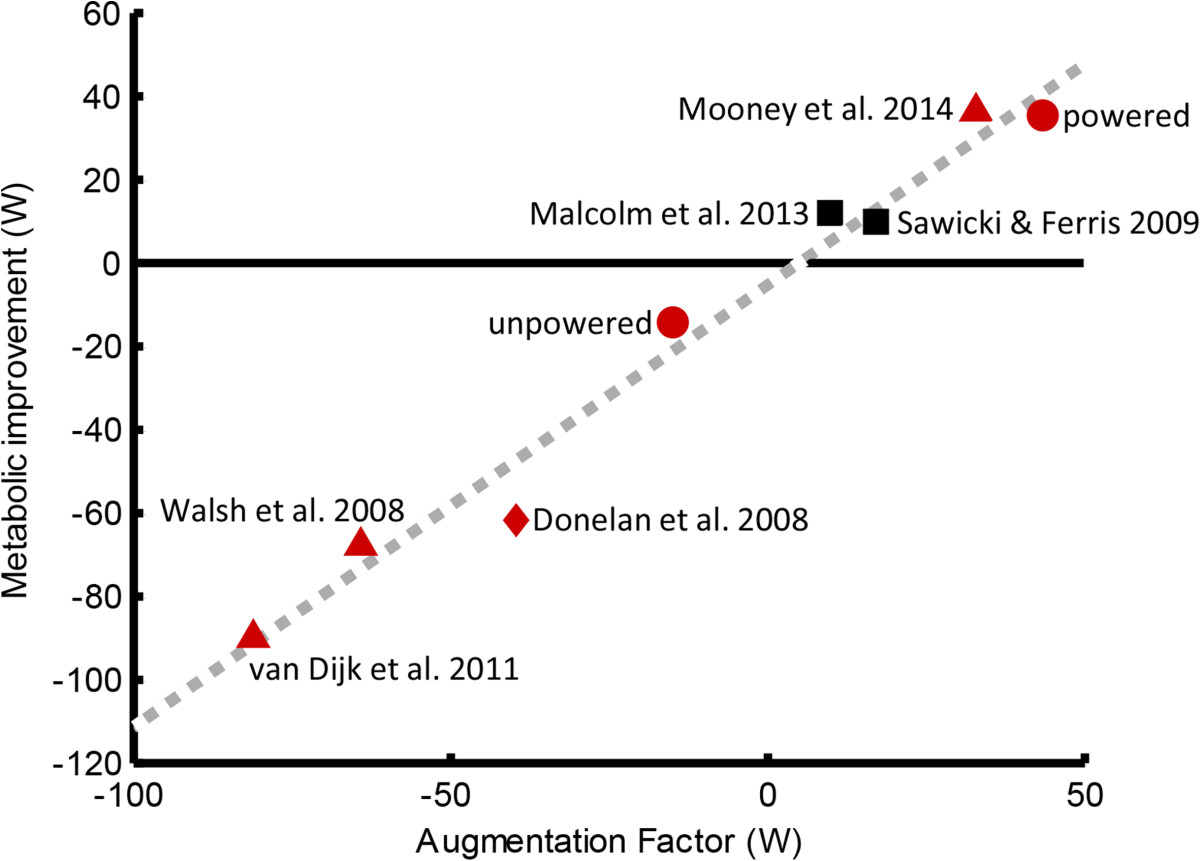
**Augmentation Factor.** The AF was calculated for six exoskeletal designs [3, 6, 10–12] and one energy harvesting design [13]. Red triangle markers are previously published studies on autonomous exoskeletons, the red diamond is an energy harvesting device, black squares are previously published studies on tethered exoskeletons, and the two red circles are the exoskeletal design of the present study. The red circle with a positive AF (powered) denotes the present exoskeleton when powered on, and the red circle with a negative AF (unpowered) denotes the present exoskeleton when the device was powered off and thus delivered zero mechanical power. The equation estimated by linear regression is *y* = 1.1*x* – 5 with an R^2^ of 0.98.

The Augmentation Factor (AF) for unloaded walking was calculated for both powered and unpowered walking with the exoskeleton. The AF predicted a metabolic reduction of 44 W while wearing the powered exoskeleton, and a metabolic increase of 15 W while wearing the unpowered exoskeleton. These new data points are compared to previous exoskeletal studies in Figure 4[3, 6, 10–13].

## Conclusion

In this study, an autonomous ankle exoskeleton was shown to reduce the metabolic cost of level walking. Compared to our previous design [6], the present exoskeleton includes a modified transmission ratio and shorter struts that resulted in an exoskeletal mass reduction of 363 g. The metabolic results align with the device’s AF, strengthening this metric as a tool for researchers. In the development of leg exoskeletons designed to reduce walking metabolism, we feel minimizing exoskeletal power dissipation and added limb mass, while providing substantial positive power, are of paramount importance.

## Endnote

^a^One experimental participant walked for only 12 minutes (instead of 20 minutes) with the exoskeleton powered on due to a device malfunction.

## Authors’ original submitted files for images

Below are the links to the authors’ original submitted files for images. Authors’ original file for figure 1 Authors’ original file for figure 2 Authors’ original file for figure 3 Authors’ original file for figure 4
