# Supplementary figures and images for: Autonomous exoskeleton reduces metabolic cost of human walking
Source: J Neuroeng Rehabil. 2014 Nov 3;11:151. doi: 10.1186/1743-0003-11-151 (PMC4236484; doi:10.1186/1743-0003-11-151)

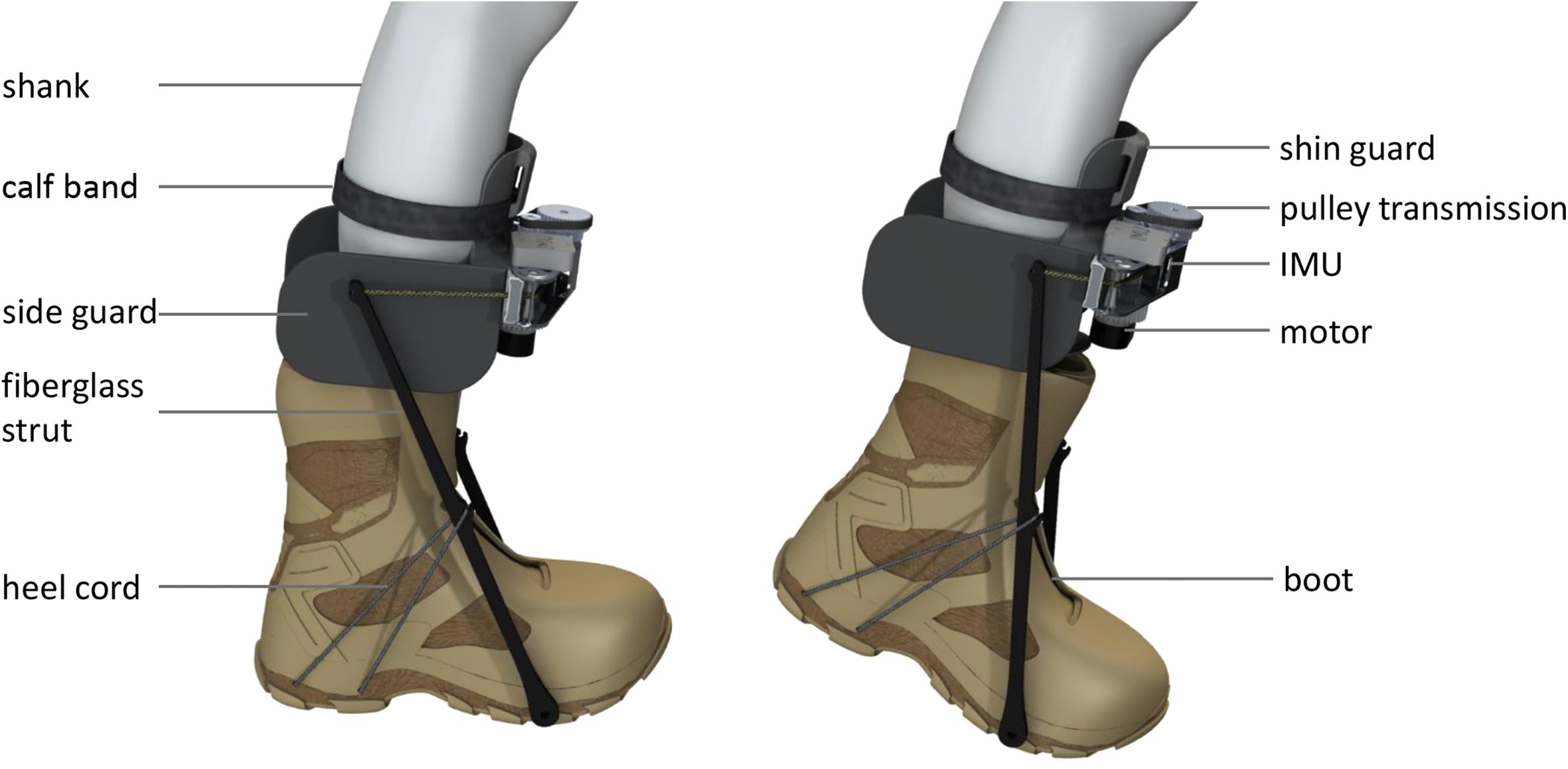

Supplement: Supplementary file 1 — Authors’ original file for figure 1 [file 12984_2014_668_MOESM1_ESM.tif]

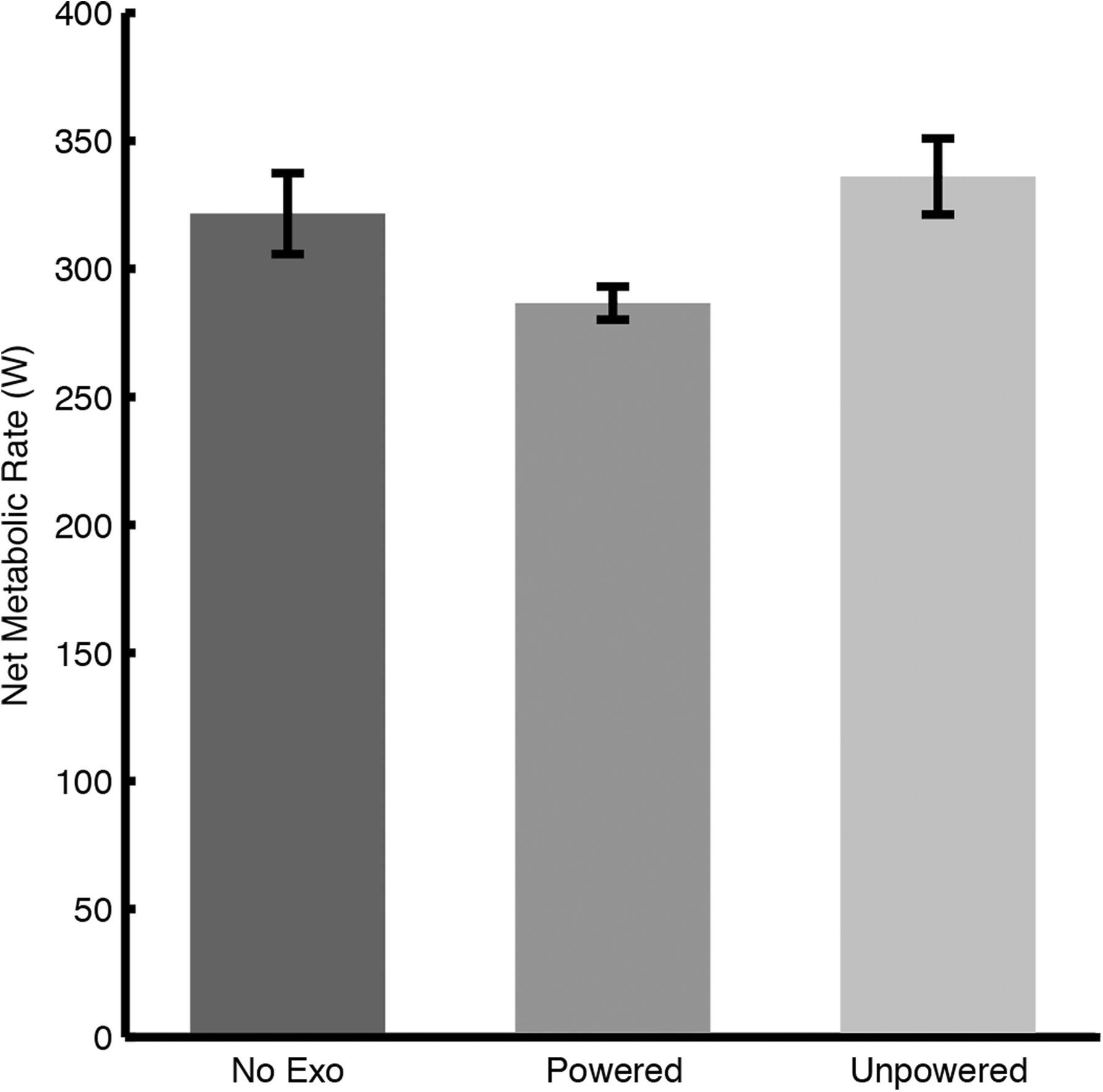

Supplement: Supplementary file 2 — Authors’ original file for figure 2 [file 12984_2014_668_MOESM2_ESM.tif]

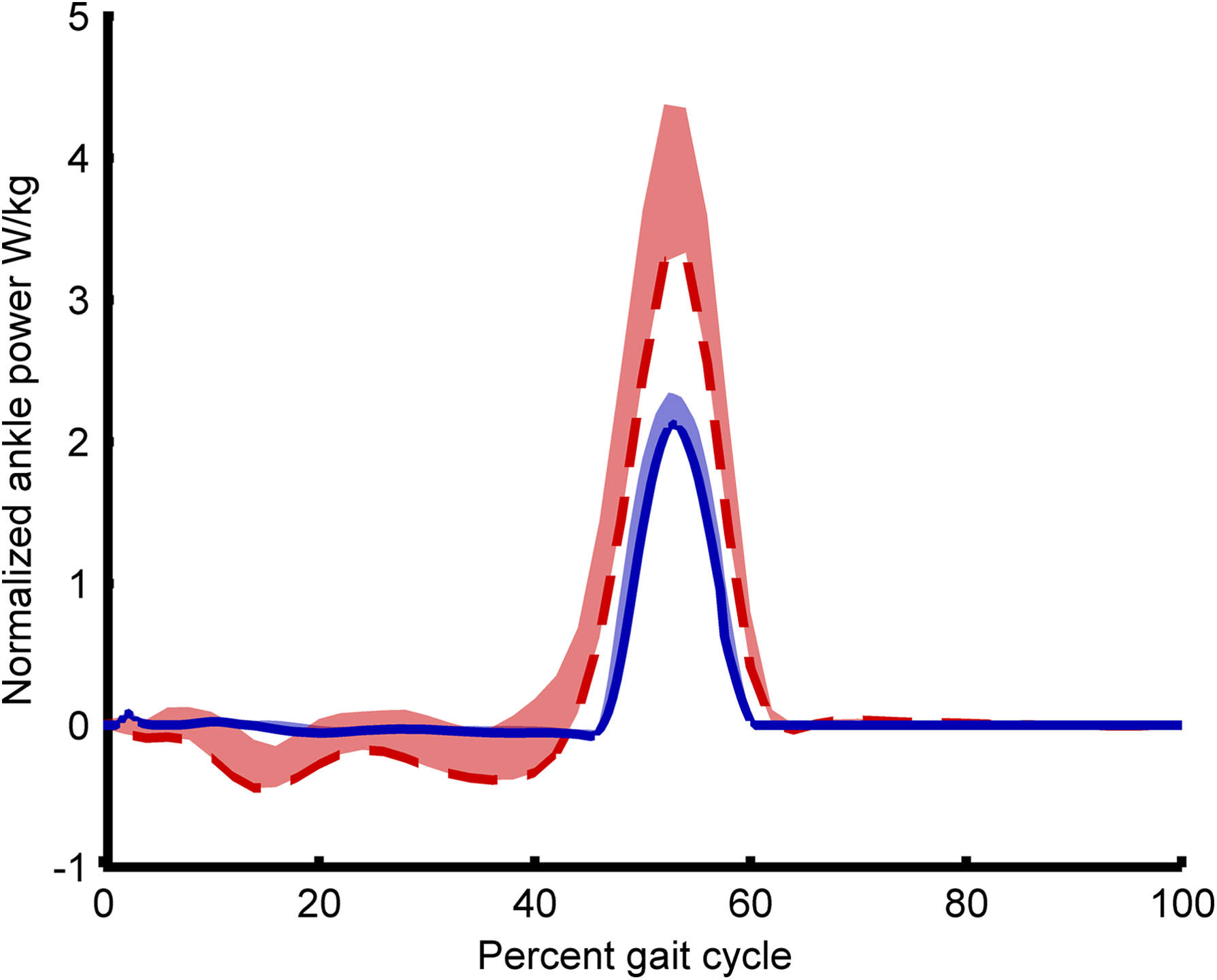

Supplement: Supplementary file 3 — Authors’ original file for figure 3 [file 12984_2014_668_MOESM3_ESM.tif]

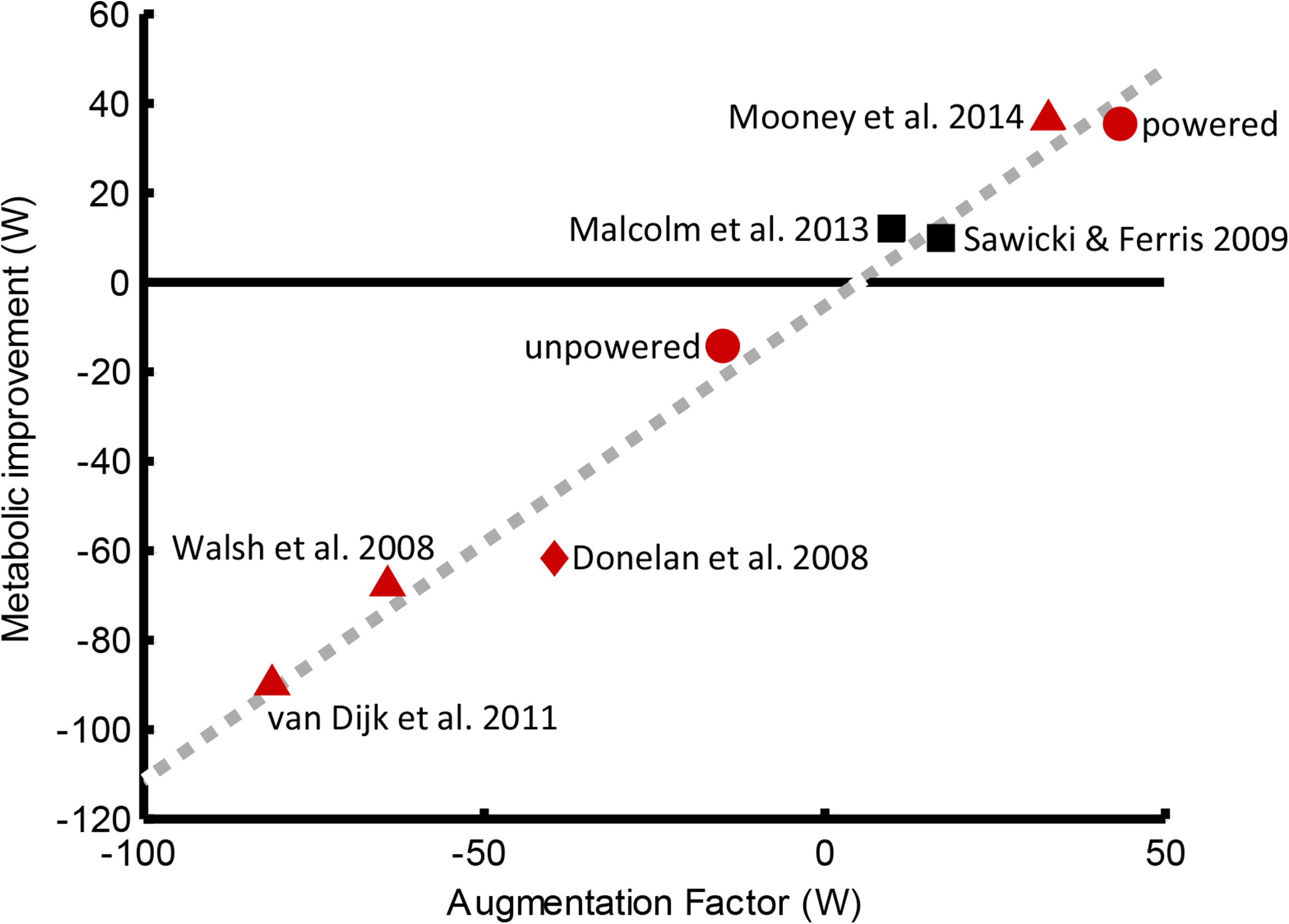

Supplement: Supplementary file 4 — Authors’ original file for figure 4 [file 12984_2014_668_MOESM4_ESM.tif]
